# Magnetic Resonance Imaging Derived Cartilage Morphological Changes and their Correlation with Patient-Reported Outcome Measures Following Knee Joint Distraction for Osteoarthritis: A 12-Month Cohort Study

**DOI:** 10.1177/19476035251357836

**Published:** 2025-08-04

**Authors:** Beth Lineham, Ahmad Joumah, Thomas Hamilton, Nagitha Wijayathunga, Hemant Pandit

**Affiliations:** 1 Leeds Institute of Rheumatic and Musculoskeletal Medicine, Leeds, UK; 2 NIHR Leeds Biomedical Research Centre, Leeds, UK; 3 Leeds Teaching Hospitals NHS Foundation Trust, Leeds, UK; 4 Nuffield Department of Orthopaedics, Rheumatology and Musculoskeletal Sciences, Oxford, UK; 5 Institute of Medical and Biological Engineering, University of Leeds, Leeds, UK

**Keywords:** knee osteoarthritis, knee joint distraction, magnetic resonance imaging, patient outcomes, cartilage morphology

## Abstract

**Aims:**

Knee osteoarthritis (OA) is a significant source of morbidity and socioeconomic burden, exacerbated by aging populations and rising body mass index. Total Knee Replacement (TKR) is effective but may result in dissatisfaction or revision, particularly in young patients. Knee Joint Distraction (KJD) offers a joint-preserving alternative that may delay or avoid replacement. This study assessed cartilage morphology changes using magnetic resonance imaging (MRI) of patients up to 1-year post-KJD in patients from a randomized controlled trial (RCT). The primary aim was to evaluate cartilage volumes at 12 months post-KJD. Secondary aims were to evaluate additional MRI parameters for cartilage morphology and compare the MRI parameters with Patient-Reported Outcome Measure (PROM) scores at 3 and 12 months.

**Methods:**

A subset of participants from an RCT comparing TKR and KJD were analyzed. The MRI and PROMs, including Knee Injury & Osteoarthritis Outcomes Score (KOOS), Oxford Knee Score (OKS), and pain visual analogue scale (VAS), were collected at baseline, 3 months, and 12 months postintervention. Cartilage segmentation using commercial software and grading using the MRI Osteoarthritis Knee Score (MOAKS) were performed.

**Results:**

Ten patients were included. Increases in mean cartilage volume were observed in all regions except the trochlear at both follow-ups. Mean cartilage thickness increased in all areas except the lateral tibia. Mean denuded bone area decreased in all regions at 12 months and in the lateral femur at 3 months. Baseline cartilage status was predictive of treatment response.

**Conclusion:**

KJD led to improvements in cartilage morphology up to 12 months, suggesting its potential as a joint-preserving strategy for knee OA. Further long-term studies are needed to confirm benefits and understand mechanisms.

## Introduction

Knee osteoarthritis (OA) causes significant morbidity and socioeconomic burden.^1^ The incidence of knee OA is increasing, partly due to aging populations, but also in younger age groups secondary to increasing population body mass index (BMI).^2^ Total Knee Replacement (TKR) is an effective treatment for end-stage OA refractory to non-surgical management. However, high rates of patient dissatisfaction are often seen.^3^ In total, 40% of all TKRs are performed in patients less than 65 years of age.^4^ Young patients face higher risks of revision surgery in their lifetime, with a lifetime revision risk of around 1 in 3 reported in patients aged under 55 years.^5^ Joint-preserving surgery is therefore preferable in certain patient groups, to enable patients (particularly, the young and active) to delay or prevent the need for irreversible joint-sacrificing operations like TKR, leading to reduced revision burden.

Knee joint distraction (KJD) is a minimally invasive joint-preserving surgery for tibiofemoral knee OA. Static, linear, external fixation is used to distract the joint over a period of 6 weeks to offload the cartilage, allowing for regeneration. It has been shown to delay the need for TKR and is associated with significant improvement in patient symptoms and function.^6,7^ The precise biological mechanism of KJD for knee OA treatment is not yet fully understood. It appears to cause a move from catabolic to anabolic pathways to enable endogenous cartilage repair mechanisms to occur. It is theorized that the process of regenerating damaged cartilage occurs in response to the changes in pressure and fluid flow within the joint space, with modulation of both the mechanical and biological environment leading to increased stem cell adherence to damaged cartilage^8^ and increased proteoglycan synthesis.^9^

The clinical effectiveness of KJD has previously been investigated through randomized controlled trials (RCTs) which have reported equivalent mid-term clinical improvements in OA patients compared to TKR and separately to high tibial osteotomy (HTO).^10,11^ In addition to improvements in PROMs, KJD has been reported to be associated with improvements in radiological parameters in both cartilage and subchondral bone^12^ with improvements lasting up to 10 years,^6^ allowing TKR to be postponed for over 10 years in two thirds of patients.^13^ Magnetic resonance imaging (MRI) has demonstrated structural recovery with increased cartilage thickness and decreased denuded subchondral bone.^14^ Subchondral bone changes have also been demonstrated with reduced cystic change and an increase in bone density following KJD.^15^ These trials, however, are small and were performed by a single developer center. At the time of writing, KJD is not currently widely used in clinical practice. In view of this, the National Institute for Health and Care Research (NIHR), UK, funded the Knee Arthroplasty versus Joint Distraction Study (KARDS), an RCT comparing the clinical and radiological outcomes of KJD vs TKR in patients with OA. The protocol has been reported previously.^16^

This study examines cartilage morphology changes following KJD in a subset of patients recruited in the KARDS RCT. Its primary aim is to evaluate cartilage volumes at 12 months post-KJD, and its secondary aim is to evaluate additional MRI parameters for cartilage morphology and compare MRI parameters with Patient-Reported Outcome Measure (PROM) scores at 3 and 12 months postintervention.

## Methods

This study constitutes a subset of participants derived from a 2-arm randomized controlled non-inferiority trial (RCT) comparing TKR and KJD, which was conducted at a single center from 2021 to 2023. The RCT was funded by the NIHR and received national ethics approval from the Yorkshire & The Humber Leeds East Research Ethics Committee. Full details of the proposed trial design were published as a protocol.^16^ Inclusion criteria included individuals requiring unicompartmental or TKR, aged 65 years or younger, with no significant alignment correction required and intact collateral knee ligaments. Exclusions comprised a severe knee deformity (>10 degrees flexion), insufficient bone density to support pins as determined by the operating surgeon, inflammatory arthritis, prior joint replacement in any limb, recent knee surgery (<6 months), prior KJD, weight exceeding 120 kg, pregnancy, lactation, or active cancer. Limb alignment was assessed via clinical examination and standard knee radiographs. The primary outcome measure was the Knee Injury & Osteoarthritis Outcomes Score (KOOS) pain score within 12 months from surgery. This is a validated questionnaire for patients with knee OA or injury.^17^ Participants also completed other components of the KOOS score, a pain visual analogue scale (VAS)^18^ and the Oxford Knee Score (OKS)^19^ at baseline, 3 months, and 12 months postsurgery.

Participation in this study was extended to all patients who provided consent for the main RCT. Randomization was conducted 6 weeks prior to surgery to allow sufficient time for patient preparation and to ensure clinical equipoise. Informed consent was obtained before randomization to either TKR or KJD, using an automated secure randomization service. No patients withdrew their consent subsequent to randomization. Surgery and perioperative management for KJD were standardized and are published previously.^16^ A definitive external fixator was used to achieve controlled linear distraction of 5 mm across the mechanical axis. The distraction device was worn for 6 weeks and subsequently removed under general anesthesia.

The KJD participants underwent baseline MRI using an 18-Channel Knee Coil in a 3.0T MR scanner (Siemens MAGNETOM Vida, Germany), with further scans at 3 and 12 months postframe removal. The MRI sequences included 3D-SPACE, VIBE, and Turbo Spin Echo with T1, T2, and proton density contrast weighting. All sequences included fat saturation to enhance the contrast of cartilage against surrounding tissues. Cartilage morphology (volume, surface area, thickness, denuded bone area) was assessed using Simpleware ScanIP software (Synopsys, CA, USA) using the image data sets from MRI. The resolution of the image data was 0.3 × 0.3 × 0.3 mm. Two independent, blinded assessors evaluated MRI data and applied the MRI Osteoarthritis Knee Score (MOAKS) scoring system.^20^ The MOAKS evaluates features relevant to the pathophysiology of OA, including bone marrow lesions, osteophytes, menisci, anterior and posterior cruciate ligaments, synovitis, effusion, and soft tissue cysts or bursitis.

Statistical analysis was conducted using SPSS v.22 software (IBM, Armonk, New York). Cartilage parameters were reported as mean ± SD or median (range) based on normality testing and *n* = 10 sample size. Only descriptive statistics are reported due to sample size. Interobserver correlation was assessed using the intraclass correlation coefficient (ICC) for 2 observers. Quantitative values derived from MRI for cartilage volume, surface area, maximum thickness, and denuded bone area were correlated with the KOOS score components, pain VAS score, and OKS score at baseline and at 3 and 12 months using Pearson’s correlation coefficient. The correlation of quantitative values derived from MRI for cartilage volume, surface area, maximum thickness, and denuded bone area at baseline with the respective values at 3 and 12 months was assessed to ascertain if baseline values had predictive value.

## Results

### Demographic Characteristics

A total of 11 patients were enrolled in the parent study and randomized to the KJD group, with 10 of these patients consenting to participate in this imaging substudy. The median age was 61.4 (interquartile range [IQR] = 56.2, 62.3) years with 7 men and 3 women. The BMI had a median of 27.9 (IQR = 26.5, 31). All patients had limb alignment within normal limits, and all had arthritis severity of 2 to 4 Kellgren-Lawrence grade. Two patients experienced pin-site infections managed with oral antibiotics. Notably, 1 patient was excluded at the 12-month mark due to the presence of metalwork in the knee following a fracture 3 months following frame removal. The data met the criteria for normality, and the results are therefore presented as means with their corresponding standard deviations.

### Cartilage Volume

**Figure 1.**
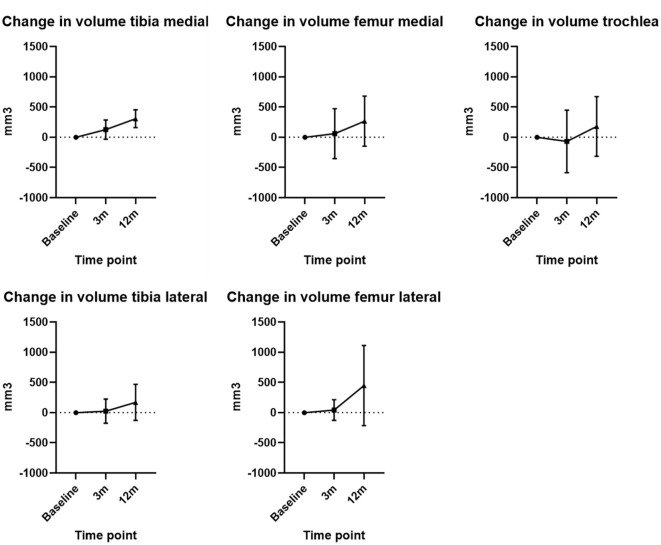
Mean cartilage volume change from baseline at 3 and 12 months in different compartments. Error bars indicate the standard deviation.

The ICC was 0.97. The evaluation of cartilage volume changes at 3 months indicated a mean increase in the tibia of 74.7 mm^3^ (SD ± 182.8 mm^3^) and femur of 11.0 mm^3^ (SD ± 383.5 mm^3^). At the 12-month assessment, there was a mean increase from baseline in the tibia of 237.6 mm^3^ (SD ± 238.9 mm^3^), and femur of 296.0 mm^3^ (SD ± 519.1 mm^3^). Changes for specific compartments are presented in **Figure 1**.

### Cartilage Maximum Thickness

**Figure 2.**
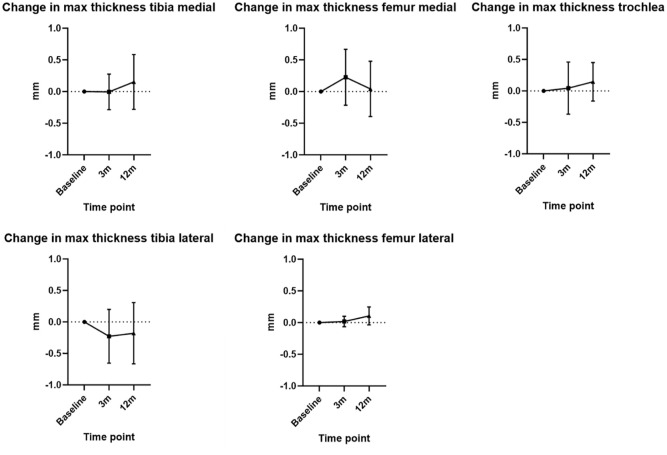
Mean change in maximum thickness from baseline to 3 and 12 months in different compartments. Mean values plotted with error bars indicating standard deviation.

The ICC was 0.99. For the tibial cartilage maximum thickness, there was a mean decrease of −0.1 mm (SD ± 0.4 mm) and −0.01 mm (SD ± 0.5 mm) at 3 months and 12 months, respectively. In contrast, the femoral cartilage maximum thickness indicated a mean increase of 0.1 mm (SD ± 0.4 mm) at 3 months and 0.1 mm (SD ± 0.3 mm) at 12 months. Changes for specific compartments are presented in **Figure 2**.

### Denuded Bone Area

**Figure 3.**
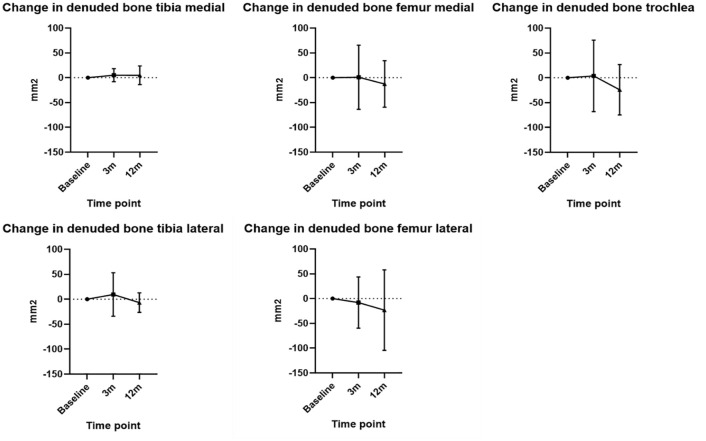
Mean denuded bone area change from baseline to 3 and 12 months in different compartments. Mean values plotted with error bars indicating standard deviation.

The ICC was 0.56. Denuded bone area increased at 3 months in the tibia by mean 7.2 mm^2^ (SD ± 30.6 mm^2^) and decreased in the femur by −1.1 mm^2^ (SD ± 60.97 mm^2^). At 12 months, there was a decrease in denuded bone area in the tibia by mean −0.6 mm^2^ (SD ± 19.5 mm^2^) and the femur of −19.9 mm^2^ (SD ± 59.1 mm^2^). The change in the denuded bone area for specific compartments is presented in **Figure 3**.

### Cartilage Surface Area

**Figure 4.**
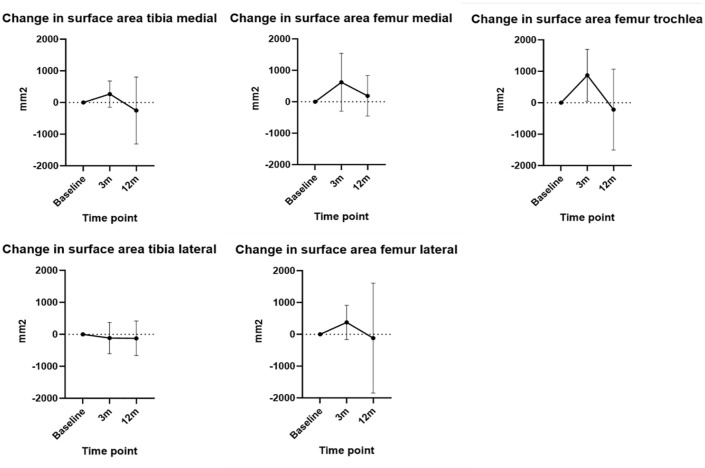
Mean cartilage surface area change from baseline to 3 and 12 months in different compartments. Mean values plotted with error bars indicating standard deviation.

The ICC was 0.99. At 3 months, there was a mean increase in cartilage surface area of 87.9 mm (SD ± 481.0 mm) in the tibia and of 623 mm (SD ± 776.6 mm) in the femur. At 12 months, a mean decrease of −192.0 mm (SD ± 829.7 mm) in the tibia and −49.2 mm (SD ± 1,254 mm) in the femur was observed. The change for specific compartments is presented in **Figure 4**.

### Correlation with Baseline

Cartilage volume values showed positive correlation with baseline to 3 months (r = .94, *P* = 0.0001), 3 months to 12 months (r = .80, *P* = 0.16), and baseline to 12 months (r = .83, *P* = 0.01).

Cartilage surface area absolute values showed positive correlations of baseline to 3 months (r = .81, *P* = 0.0001), 3 months to 12 months (r= .88, *P* = 0.0004), and baseline to 12 months (r = .85, *P* = 0.007).

Maximum cartilage thickness demonstrated a strong positive correlation at baseline to 3 months (r = .82, *P* = 0.01) and a positive correlation from 3 months to 12 months (r = .64, *P* = 0.28) and baseline to 12 months (r = .43, *P* = 0.28).

Denuded bone area did not demonstrate correlation at baseline to 3 months (r = .21, *P* = 0.61), 3 months to 12 months (r = −.01, *P* = 0.98), or baseline to 12 months (r = .30, *P* = 0.46).

### Magnetic Resonance Imaging Osteoarthritis Knee Score

The ICC was 0.85. The median (& range) of baseline MOAKS scores was 12 (range = 5-36) for bone marrow lesions, 27 (22-49) for cartilage damage, and 15 (1-22) for osteophytes. At the 3-month follow-up, median percentage change in MOAKS scores was 20.4% (−58.3 to 350%) for bone marrow lesions, 0.0% (−49.0 to 30.8%) for cartilage damage, and −3.3% (−25.0 to 100.0%) for osteophytes. While at 12 months, the median percentage change from baseline scores was −20.8% (−100.0 to 240.0%) for bone marrow lesions, 13.6% (−100.0 to 46.2%) for cartilage damage, and 5.7% (−100.0 to 100.0%) for osteophytes. All components of the MOAKS score are outlined in **Table 1**.

**Table 1.** Median MOAKS Component Score Percentage Change From Baseline to 3 Months and 12 Months Follow-up.

	Bone marrow lesions	Cartilage	Osteophytes
Baseline score	12	27	15
% change at 3 months	20.4%	0.0%	-3.3%
% change at 12 months	-20.8%	-13.6%	5.7%

### Knee Injury & Osteoarthritis Outcomes Score Pain Correlation

Median KOOS pain score at baseline was 40.5. The KOOS pain score increased at 3 months to a median of 50 and at 12 months to median of 56. The KOOS pain scores were not available for 2 patients at 12 months. There were weak positive correlations of median KOOS pain scores with cartilage surface area (r =.38, *P* = 0.205), cartilage volume (r = .34, *P* = 0.08), denuded bone area (r = .15, *P* = 0.47), and maximum cartilage thickness (r = .01, *P* = 0.62), although none of these reached statistical significance.

### Visual Analogue Scale Pain Correlation

Median VAS pain score at baseline was 5.5. At 3 months and 12 months, VAS pain scores decreased to 5 and 3.75, respectively. There were weak negative correlations of median VAS pain scores with cartilage surface area (r = −.32, *P* = 0.13), cartilage volume (r = −.30, *P* = 0.13), denuded bone area (r = −.18, *P* = 0.40), and maximum cartilage thickness (r = −.23, *P* = 0.25), although none of these reached statistical significance.

### OKS Correlation

Median OKS at baseline was 18. At 3 months, median OKS decreased slightly to 17.5 and increased at 12 months to 30. There were moderate positive correlations of median OKS with cartilage surface area (r = .44, *P* = 0.02) and cartilage volume (r = .45, *P* = 0.02), which were both statistically significant. There was no significant correlation of OKS with denuded bone area (r = .04, *P* = 0.08) or maximum cartilage thickness (r = .25, *P* = 0.21).

## Discussion

This study elucidates the impact of KJD on cartilage morphology as assessed through MRI parameters at 3 and 12 months postintervention. Modest improvements in cartilage MRI morphological parameters following KJD were observed at 3 and 12 months. However, MOAKS scores showed deterioration in OA features such as osteophytes, highlighting ongoing disease progression despite cartilage improvements.

The selection of MRI-based morphological parameters in this study was guided by their established application in prior research. Cartilage volume quantification via MRI has been validated extensively through comparisons with cadaveric and surgical specimens^21-24^ and computed tomography (CT) arthrography.^25^ Additional studies have demonstrated the validity of MRI-derived cartilage thickness measurements as reliable markers of cartilage status.^25,26^ Similarly, the identification of denuded bone or full-thickness cartilage defects has been corroborated through direct visualization of cartilage lesions during arthroscopy.^27-29^

The observed increases in cartilage thickness and reductions in denuded bone area at 12 months are consistent with previous KJD studies that have demonstrated similar trends in cartilage thickness and denuded bone area at 1-year postintervention.^12^ It is important to highlight that there have been no other published reports on MRI changes prior to 12 months following KJD. Based on natural history studies, a worsening of cartilage volume and thickness on MRI is typically expected in knee OA, with more pronounced changes observed in cases of advanced OA.^30^

The observed improvements in trochlear cartilage, which have not been previously reported, suggest potential benefits for patellofemoral OA, expanding the potential indications for the procedure. These improvements may be attributable to the offloading of the patella when the leg is maintained in extension, as the patella does not engage with the trochlea until approximately 20 to 30 degrees of knee flexion.^31^ In addition, biological factors may play a role; changes in the synovial fluid environment, such as reduced synovial fluid density during distraction, have been shown in laboratory settings to enhance mesenchymal stem cell adhesion to cartilage surfaces.^8^ This has important implications for clinical decision-making and patient counseling.

The clinical relevance of MRI-based morphological parameters has been previously explored, although results have been variable.^32-34^ Correlations between MRI metrics, or even radiographic features, and clinical symptoms such as pain and function are inconsistent. In addition, these imaging parameters are subject to significant variability, as they may be influenced by multiple confounding factors. For instance, both cartilage volume and thickness can be affected by the patient’s recent physical activity^35^ and may appear increased in early stages of OA due to cartilage swelling.^36^ Nevertheless, several studies have demonstrated associations between MRI-assessed cartilage morphology and PROMs in the knee.^6,37,38^ In contrast, denuded bone or full-thickness cartilage loss has not shown a consistent relationship with clinical symptom severity.^39^ In this study, considerable variation in the measured MRI parameters was observed. Cartilage surface area changes had particularly high variation between participants but have not been previously reported following cartilage regeneration procedures, precluding direct comparisons. The significance of changes in cartilage surface area is unclear, as decreased smoothness (higher values of cartilage surface area) has been shown as a marker for OA.^40^ However, an increase in cartilage surface area may also be related to increased cartilage volume. The changes in cartilage volume have been documented by van der Woude *et al*.^10^ In the KJD vs. HTO trial, there was a similar variation in volume changes to our findings in this study. However, it is challenging to directly compare these findings due to potential differences in MRI protocols and analyses, despite similar methodology. Despite the variation, the high ICC values in this study lend confidence to the accuracy of the MRI analysis.

The robust positive correlation observed between baseline values for cartilage volume and surface area, and their corresponding values at 3 and 12 months, highlights the predictive utility of initial cartilage status in determining treatment response. This suggests that individuals with greater cartilage reserves at the outset showed more pronounced improvements, while those who exhibited early improvement tended to sustain and build upon it over time. In contrast, denuded bone volume showed no correlation.

However, this study had certain limitations. The sample size is relatively small at only 10 patients. One group has conducted a larger study in 2011,^12^ involving 20 patients without a control group, with outcomes up to 10 years.^6^ Importantly, this study represents the largest clinical series on KJD outside of this one group in the Netherlands and the first such investigation in the United Kingdom. This study did not include the 12-month scan of 1 participant due to a femoral fracture necessitating metalwork in the knee, which compromised MRI segmentation accuracy and precluded analysis at the 12-month mark.

The technical accuracy (the accuracy by which the MRI parameter is measured) of MRI-based cartilage morphological measurements has been evaluated in different studies, and most of these have reported high agreement between MRI-based quantification and the experimental/alternative method of quantification for validation, with random errors of approximately 5% to 10%.^41^

However, accurate segmentation of cartilage from surrounding tissue in the image data is necessary to derive reliable quantitative data for cartilage morphological parameters, particularly when temporal changes in cartilage morphology are of significant importance, such as in longitudinal studies. Since the thickness of articular cartilage in healthy knees is only up to 3 mm approximately, and even thinner in knees with OA, high image spatial resolutions were employed for the MRI in this study in order to support segmentation accuracy. In addition, the use of 3D MRI sequences allowed much thinner slice thicknesses (isotropic voxels), helping to reduce the partial volume–related errors in segmentation.

In this study, there was no control group of patients with OA who underwent no intervention. However, longitudinal observational studies have demonstrated that individuals with Kellgren-Lawrence grade >2 typically exhibit progressive structural deterioration, with an average cartilage volume loss of 107.9 mm^3^/year.^42^ A previous study comparing KJD patients with OA patients who had no surgical intervention noted statistically significant beneficial changes up to 2 years but not significant at 5 years.^43^

The PROM scores, even though slightly worsened at 3 months, all showed improvement at 12 months. While detailed PROM analyses are presented in the parent KARDS trial, the 12-month KOOS and VAS pain scores observed here are consistent with prior studies of KJD,^12^ supporting the reproducibility of outcomes across cohorts. Notably, there was no strong correlation between PROM scores and the changes observed on MRI. The OKS score did have moderate positive correlations that were statistically significant, but KOOS scores and VAS pain scores did not correlate significantly. This underscores the previously reported issue that cartilage changes do not always correlate with PROM scores and that the nature of symptoms in OA does not entirely align with imaging results.^32,44^ It is likely more important to consider the overall progression of a patient’s condition rather than relying on a single snapshot from imaging or PROM scores. While the 3- and 12-month follow-up periods provide valuable early insights, they are too early to draw definitive conclusions about the long-term effectiveness of KJD. However, previous studies have demonstrated that early improvements tend to correlate with sustained cartilage regeneration and symptom relief at 5 and even 10 years.^45^ Our findings appear to align with this pattern, but longer-term follow-up is essential to determine the durability of these effects and to better inform clinical decision-making.^45^

## Conclusion

This study demonstrates improvements in cartilage MRI morphological parameters at 3 and 12 months following the emerging joint-sparing technique of KJD. Therefore, KJD has the potential to be considered as a surgical option for young patients with severe knee OA. However, variation in results was considerably high, and longer-term follow-up with a larger patient sample is required in addition to correlation with clinical outcomes and biomarkers, to fully understand clinical outcomes and the mechanism of action of cartilage regeneration following KJD.

## References

[1] Patel A Pavlou G Mújica-Mota RE Toms AD . The epidemiology of revision total knee and hip arthroplasty in England and Wales: a comparative analysis with projections for the United States. Bone Joint J. 2015;97-B(8):1076-81. 10.1302/0301-620X.97B8.35170 26224824

[2] Wallace IJ Worthington S Felson DT Jurmain RD Wren KT Maijanen H , et al. Knee osteoarthritis has doubled in prevalence since the mid-20th century. Proc Natl Acad Sci U S A. 2017;114(35):9332-6. 10.1073/pnas.1703856114 PMC5584421 28808025

[3] DeFrance MJ Scuderi GR . Are 20% of patients actually dissatisfied following total knee arthroplasty? A systematic review of the literature. J Arthroplasty. 2023;38(3):594-9. 10.1016/j.arth.2022.10.011 36252743

[4] Kurtz SM Lau E Ong K Zhao K Kelly M Bozic KJ . Future young patient demand for primary and revision joint replacement: national projections from 2010 to 2030. Clin Orthop Relat Res. 2009;467(10):2606-12. 10.1007/s11999-009-0834-6 PMC2745453 19360453

[5] Walker-Santiago R Tegethoff JD Ralston WM Keeney JA . Revision total knee arthroplasty in young patients: higher early reoperation and rerevision. J Arthroplasty. 2021;36(2):653-6. 10.1016/j.arth.2020.08.052 32948426

[6] Jansen MP Mastbergen SC MacKay JW Turmezei TD Lafeber F . Knee joint distraction results in MRI cartilage thickness increase up to 10 years after treatment. Rheumatology (Oxford). 2022;61(3):974-82. 10.1093/rheumatology/keab456 PMC8889280 34022055

[7] Jansen MP Boymans TAEJ Custers RJH Van Geenen RCI Van Heerwaarden RJ Huizinga MR , et al. Knee joint distraction as treatment for osteoarthritis results in clinical and structural benefit: a systematic review and meta-analysis of the limited number of studies and patients available. Cartilage. 2021;13(1 Suppl):1113S-1123S. 10.1177/1947603520942945 PMC8808886 32698704

[8] Baboolal TG Mastbergen SC Jones E Calder SJ Lafeber FP McGonagle D . Synovial fluid hyaluronan mediates MSC attachment to cartilage, a potential novel mechanism contributing to cartilage repair in osteoarthritis using knee joint distraction. Ann Rheum Dis. 2016;75(5):908-15. 10.1136/annrheumdis-2014-206847 PMC4853581 25948596

[9] Lafeber F Veldhuijzen JP Vanroy JL Huber-Bruning O Bijlsma JW . Intermittent hydrostatic compressive force stimulates exclusively the proteoglycan synthesis of osteoarthritic human cartilage. Br J Rheumatol. 1992;31(7):437-42. 10.1093/rheumatology/31.7.437 1628164

[10] van der Woude JAD Wiegant K van Heerwaarden RJ Spruijt S van Roermund PM Custers RJH , et al. Knee joint distraction compared with high tibial osteotomy: a randomized controlled trial. Knee Surg Sports Traumatol Arthrosc. 2017;25(3):876-86. 10.1007/s00167-016-4131-0 PMC5332499 27106926

[11] van der Woude JA Wiegant K van Heerwaarden RJ Spruijt S Emans PJ Mastbergen SC , et al. Knee joint distraction compared with total knee arthroplasty: a randomised controlled trial. Bone Joint J. 2017;99-B(1):51-8. 10.1302/0301-620X.99B1.BJJ-2016-0099.R3 28053257

[12] Intema F Van Roermund PM Marijnissen AC Cotofana S Eckstein F Castelein RM , et al. Tissue structure modification in knee osteoarthritis by use of joint distraction: an open 1-year pilot study. Ann Rheum Dis. 2011;70(8):1441-6. 10.1136/ard.2010.142364 PMC3128325 21565898

[13] Wiegant K van Roermund P van Heerwaarden R Spruijt S Custers R Kuchuk N , et al. Total knee prosthesis after knee joint distraction treatment. J Surg Res. 2015;1(3):066-71.

[14] Takahashi T Baboolal TG Lamb J Hamilton TW Pandit HG . Is knee joint distraction a viable treatment option for knee OA?-a literature review and meta-analysis. J Knee Surg. 2019;32(8):788-95. 10.1055/s-0038-1669447 30157528

[15] Chen Y Sun Y Pan X Ho K Li G . Joint distraction attenuates osteoarthritis by reducing secondary inflammation, cartilage degeneration and subchondral bone aberrant change. Osteoarthritis Cartilage. 2015;23(10):1728-35. 10.1016/j.joca.2015.05.018 26028135

[16] Tassinari CJ Higham R Smith IL Arnold S Mujica-Mota R Metcalfe A , et al. Clinical and cost-effectiveness of knee arthroplasty versus joint distraction for osteoarthritis (KARDS): protocol for a multicentre, phase III, randomised control trial. BMJ Open. 2022;12(6):e062721. 10.1136/bmjopen-2022-062721 PMC9247693 35772819

[17] Roos EM Lohmander LS . The knee injury and osteoarthritis outcome score (KOOS): from joint injury to osteoarthritis. Health Qual Life Outcomes. 2003;1:64. 14613558 10.1186/1477-7525-1-64 PMC280702

[18] Hawker GA Mian S Kendzerska T French M . Measures of adult pain: visual analog scale for pain (VAS Pain), numeric rating scale for pain (NRS Pain), McGill pain questionnaire (MPQ), short-form McGill pain questionnaire (SF-MPQ), chronic pain grade scale (CPGS), short form-36 bodily pain scale (SF-36 BPS), and measure of intermittent and constant osteoarthritis pain (ICOAP). Arthritis Care Res (Hoboken). 2011;63(Suppl 11):S240-S252. 10.1002/acr.20543 22588748

[19] Xie F Ye H Zhang Y Liu X Lei T Li SC . Extension from inpatients to outpatients: validity and reliability of the Oxford knee score in measuring health outcomes in patients with knee osteoarthritis. Int J Rheum Dis. 2011;14(2):206-10. 10.1111/j.1756-185X.2010.01580.x 21518321

[20] Hunter DJ Guermazi A Lo GH Grainger AJ Conaghan PG Boudreau RM , et al. Evolution of semi-quantitative whole joint assessment of knee OA: MOAKS (MRI osteoarthritis knee score). Osteoarthritis Cartilage. 2011;19(8):990-1002. 21645627 10.1016/j.joca.2011.05.004 PMC4058435

[21] Burgkart R Glaser C Hyhlik-Dürr A Englmeier KH Reiser M Eckstein F . Magnetic resonance imaging-based assessment of cartilage loss in severe osteoarthritis: accuracy, precision, and diagnostic value. Arthritis Rheum. 2001;44(9):2072-7. 10.1002/1529-0131(200109)44:9<2072::AID-ART357>3.0.CO;2-3 11592369

[22] Cicuttini F Forbes A Morris K Darling S Bailey M Stuckey S . Gender differences in knee cartilage volume as measured by magnetic resonance imaging. Osteoarthritis Cartilage. 1999;7(3):265-71. 10.1053/joca.1998.0200 10329301

[23] Dupuy DE Spillane RM Rosol MS Rosenthal DI Palmer WE Burke DW , et al. Quantification of articular cartilage in the knee with three-dimensional MR imaging. Acad Radiol. 1996;3(11):919-24. 10.1016/s1076-6332(96)80299-6 8959181

[24] Eckstein F Westhoff J Sittek H Maag KP Haubner M Faber S , et al. In vivo reproducibility of three-dimensional cartilage volume and thickness measurements with MR imaging. AJR Am J Roentgenol. 1998;170(3):593-7. 10.2214/ajr.170.3.9490936 9490936

[25] Eckstein F Charles HC Buck RJ Kraus VB Remmers AE Hudelmaier M , et al. Accuracy and precision of quantitative assessment of cartilage morphology by magnetic resonance imaging at 3.0T. Arthritis Rheum. 2005;52(10):3132-6. 10.1002/art.21348 16200592

[26] Kornaat PR Reeder SB Koo S Brittain JH Yu H Andriacchi TP , et al. MR imaging of articular cartilage at 1.5T and 3.0T: comparison of SPGR and SSFP sequences. Osteoarthritis Cartilage. 2005;13(4):338-44. 10.1016/j.joca.2004.12.008 15780647

[27] Bredella MA Tirman PF Peterfy CG Zarlingo M Feller JF Bost FW , et al. Accuracy of T2-weighted fast spin-echo MR imaging with fat saturation in detecting cartilage defects in the knee: comparison with arthroscopy in 130 patients. AJR Am J Roentgenol. 1999;172(4):1073-80. 10.2214/ajr.172.4.10587150 10587150

[28] Disler DG McCauley TR Kelman CG Fuchs MD Ratner LM Wirth CR , et al. Fat-suppressed three-dimensional spoiled gradient-echo MR imaging of hyaline cartilage defects in the knee: comparison with standard MR imaging and arthroscopy. AJR Am J Roentgenol. 1996;167(1):127-32. 10.2214/ajr.167.1.8659356 8659356

[29] Kijowski R Blankenbaker DG Davis KW Shinki K Kaplan LD De Smet AA . Comparison of 1.5- and 3.0-T MR imaging for evaluating the articular cartilage of the knee joint. Radiology. 2009;250(3):839-48. 10.1148/radiol.2503080822 19164121

[30] Eckstein F Maschek S Wirth W Hudelmaier M Hitzl W Wyman B , et al. One year change of knee cartilage morphology in the first release of participants from the osteoarthritis initiative progression subcohort: association with sex, body mass index, symptoms and radiographic osteoarthritis status. Ann Rheum Dis. 2009;68(5):674-9. 10.1136/ard.2008.089904 PMC2976866 18519425

[31] Loudon JK . Biomechanics and pathomechanics of the patellofemoral joint. Int J Sports Phys Ther. 2016;11(6):820-30. PMC5095937 27904787

[32] Blackman AJ Smith MV Flanigan DC Matava MJ Wright RW Brophy RH . Correlation between magnetic resonance imaging and clinical outcomes after cartilage repair surgery in the knee: a systematic review and meta-analysis. Am J Sports Med. 2013;41(6):1426-34. 10.1177/0363546513485931 23631884

[33] de Windt TS Welsch GH Brittberg M Vonk LA Marlovits S Trattnig S , et al. Is magnetic resonance imaging reliable in predicting clinical outcome after articular cartilage repair of the knee? A systematic review and meta-analysis. Am J Sports Med. 2013;41(7):1695-702. 10.1177/0363546512473258 23364897

[34] Lineham B Wijayathunga H Moran E Shuweihdi F Gupta H Pandit H , et al. A systematic review demonstrating correlation of MRI compositional parameters with clinical outcomes following articular cartilage repair interventions in the knee. Osteoarthr Cartil Open. 2023;5(3):100388. 37560388 10.1016/j.ocarto.2023.100388 PMC10407572

[35] Coburn SL Crossley KM Kemp JL Warden SJ West TJ Bruder AM , et al. Immediate and delayed effects of joint loading activities on knee and hip cartilage: a systematic review and meta-analysis. Sports Med Open. 2023;9(1):56. 37450202 10.1186/s40798-023-00602-7 PMC10348990

[36] Cipolletta E Smerilli G Mirza Mashadi R Mandl P Filippucci E . Is thickened hyaline cartilage on ultrasound a sign of osteoarthritis? A within-person, between-joint pilot study. BMC Rheumatol. 2025;9(1):24. 40012080 10.1186/s41927-025-00473-3 PMC11863393

[37] Hunter DJ March L Sambrook PN . The association of cartilage volume with knee pain. Osteoarthritis Cartilage. 2003;11(10):725-9. 10.1016/s1063-4584(03)00160-2 13129691

[38] Wluka AE Wolfe R Stuckey S Cicuttini FM . How does tibial cartilage volume relate to symptoms in subjects with knee osteoarthritis. Ann Rheum Dis. 2004;63(3):264-8. 10.1136/ard/2003.007666 PMC1754924 14962960

[39] Illingworth KD El Bitar Y Siewert K Scaife SL El-Amin S Saleh KJ . Correlation of WOMAC and KOOS scores to tibiofemoral cartilage loss on plain radiography and 3 Tesla MRI: data from the osteoarthritis initiative. Knee Surg Sports Traumatol Arthrosc. 2014;22(7):1649-58. 10.1007/s00167-013-2402-6 23338667

[40] Tummala S Bay-Jensen AC Karsdal MA Dam EB . Diagnosis of osteoarthritis by cartilage surface smoothness quantified automatically from knee MRI. Cartilage. 2011;2(1):50-9. 10.1177/1947603510381097 PMC4300790 26069569

[41] Eckstein F Cicuttini F Raynauld JP Waterton JC Peterfy C . Magnetic resonance imaging (MRI) of articular cartilage in knee osteoarthritis (OA): morphological assessment. Osteoarthritis Cartilage. 2006;14 Suppl A:A46-A75. 10.1016/j.joca.2006.02.026 16713720

[42] Cai G Cicuttini F Aitken D Laslett LL Zhu Z Winzenberg T , et al. Comparison of radiographic and MRI osteoarthritis definitions and their combination for prediction of tibial cartilage loss, knee symptoms and total knee replacement: a longitudinal study. Osteoarthritis Cartilage. 2020;28(8):1062-70. 10.1016/j.joca.2020.04.017 32413465

[43] van der Woude JAD Wiegant K van Roermund PM Intema F Custers RJH Eckstein F , et al. Five-year follow-up of knee joint distraction: clinical benefit and cartilaginous tissue repair in an open uncontrolled prospective study. Cartilage. 2017;8(3):263-71. 10.1177/1947603516665442 PMC5625862 28618871

[44] Özden F Nadiye Karaman Ö Tuğay N Yalın Kilinç C Mihriban Kilinç R Umut Tuğay B . The relationship of radiographic findings with pain, function, and quality of life in patients with knee osteoarthritis. J Clin Orthop Trauma. 2020;11(Suppl 4):S512-S517. 10.1016/j.jcot.2020.04.006 PMC7394788 32774020

[45] Jansen MP van der Weiden GS Van Roermund PM Custers RJH Mastbergen SC Lafeber FPJG . Initial tissue repair predicts long-term clinical success of knee joint distraction as treatment for knee osteoarthritis. Osteoarthritis Cartilage. 2018;26(12):1604-8. 10.1016/j.joca.2018.08.004 30138728

